# Lightning can strike twice: recurrent multiple evanescent white dot syndrome (MEWDS) following both COVID-19 vaccination and subsequent COVID-19 infection

**DOI:** 10.1186/s12348-023-00355-0

**Published:** 2023-08-24

**Authors:** Hannah W. Ng, Rachael L. Niederer

**Affiliations:** 1 The University of Auckland, Ophthalmology, Auckland, New Zealand; 2 Greenlane Clinical Centre, Ophthalmology, Auckland, New Zealand

## Abstract

Multiple evanescent white dot syndrome has been reported to occur following COVID-19 vaccination and also secondary to COVID-19 infection. Increasingly, patients are querying their risk from further vaccination against COVID-19, vaccination for other diseases (such as influenza) and the risk of disease with COVID-19 infection itself. Here we report an interesting case in which the patient developed MEWDS following COVID vaccination, then, one year later, developed MEWDS in the fellow eye associated with COVID-19 infection.

## Image essay

An otherwise healthy 28-year-old woman presented with right central scotoma, photopsia and decreased vision. Symptoms started 2 days after receiving Pfizer-BioNTech SARS-CoV-2 (COVID-19) vaccination. She denied prodromal symptoms or fever at any stage following vaccination.

**Fig. 1:**
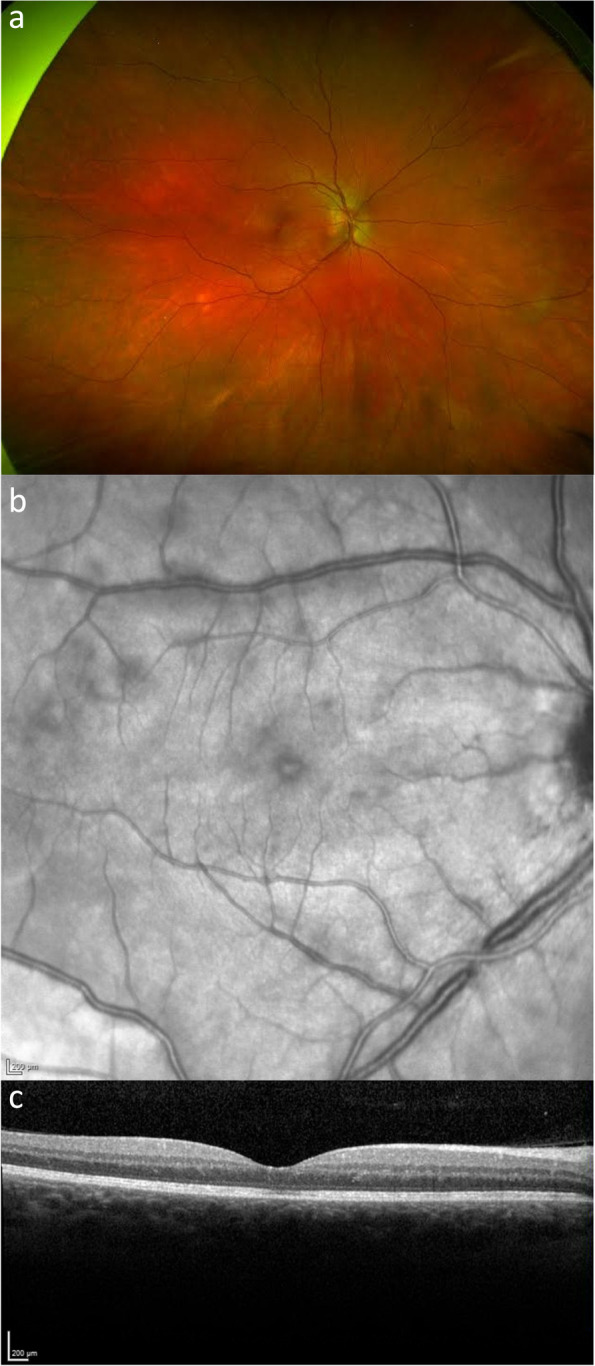
**a** Wide-field colour fundus photo **b**) Fundus infrared image of the right eye **c**) OCT macula in a patient with multiple evanescent white dot syndrome associated with COVID-19 vaccination

On examination, best corrected visual acuity was 20/50 right eye, 20/20 left eye. The anterior segment was healthy but in the right eye, 1 + anterior vitreous cells, mild optic disc oedema and multiple pale coloured lesions scattered near the posterior pole and nasal to the optic disc were observed. Fundus autofluorescence showed multiple small, hyper-auto-fluorescent lesions consistent with a diagnosis of multiple evanescent white dot syndrome (MEWDS). Optical coherence tomography (OCT) demonstrated subtle areas of disruption at the ellipsoid layer which corresponded to the white lesions seen on clinical exam and on infrared imaging (Fig. 1). Her symptoms resolved without treatment over a 3 month period with a final visual acuity of 20/20 in the affected eye. She had no ocular manifestations following the first or third COVID-19 vaccination. Routine blood workup at the time of infection including full blood count, renal function, CRP, HbA1c was negative.

**Fig. 2:**
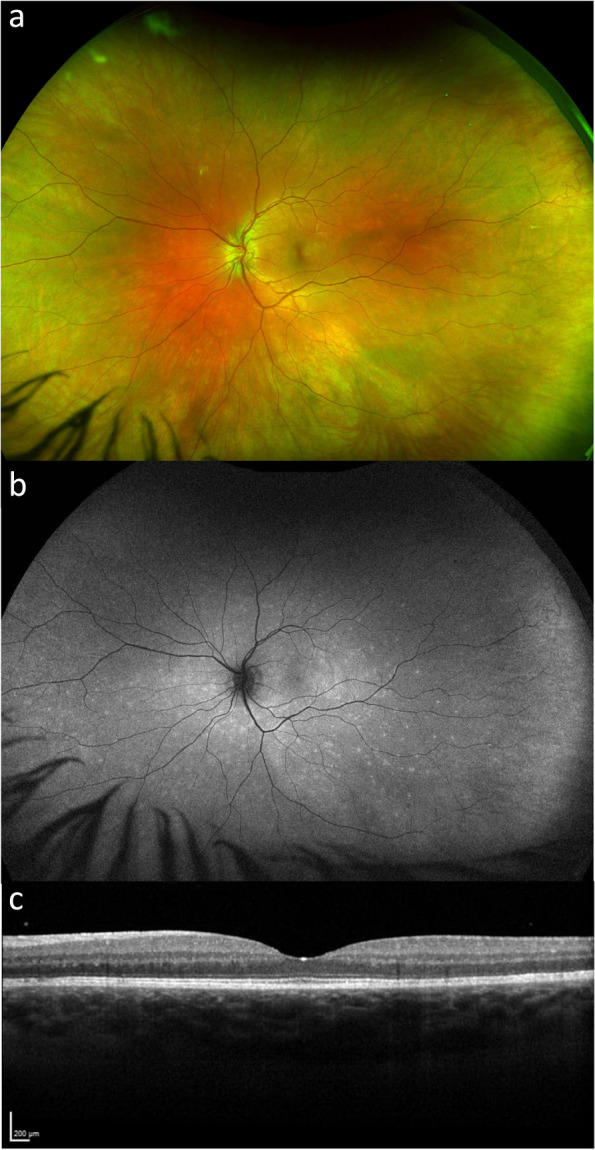
**a** Wide-field colour fundus photo and **b**) Fundus autofluorescence **c**) OCT macula of the same patient one year later with multiple evanescent white dot syndrome associated with COVID-19 infection in the contralateral left eye

One year later, the patient represented with similar symptoms in the contralateral eye and was again diagnosed with MEWDS (Fig. 2). Interestingly, seven days following symptom onset, the patient tested positive for COVID-19 infection, for which she otherwise developed mild coryzal symptoms and 2 days of fever. Workup for possible alternative diagnoses including full blood count, renal function, CRP, HbA1c, serum ACE, HLA-B27 cell surface marker, hepatitis screen and HIV were negative. Furthermore, an MRI brain was performed which was unremarkable. No hospitalization or treatment was required, and symptoms resolved over a 9 month period, with a final visual acuity of 20/25 in the affected eye. She has not had further recurrences, despite receiving the annual influenza vaccination.

MEWDS, first described in 1984, is an idiopathic inflammatory disease of the outer retina that is thought to be a transient, viral-induced autoimmune reaction [1, 2]. Though the pathogenesis is not fully understood, it has been associated with a flu-like prodrome and has also been reported following a number of vaccinations including hepatitis A, B, human papillomavirus (HPV), influenza, measles-mumps-rubella, varicella virus, rabies, yellow fever in addition to the COVID-19 vaccine [3, 4].

There have been 15 reported cases of MEWDS following COVID-19 vaccination [4] and at least 3 following COVID-19 infection. However, to the best of our knowledge, this is the first case of recurrent MEWDS following both COVID-19 vaccination and subsequent infection. It would be prudent for clinicians to monitor susceptible patients, especially those who have had uveitis following COVID-19 vaccine, to be monitored for ocular disease in the event of subsequent COVID-19 infection.

## Data Availability

Not applicable.

## References

[1] Jampol LM, Sieving PA, Pugh D, Fishman GA, Gilbert H (1984). Multiple evanescent white dot syndrome: I. Clinical findings. Arch Ophthalmol.

[2] De Salvo G, Meduri A, Vaz-Pereira S, Spencer D (2022). An uncommon cold of the retina. Surv Ophthalmol.

[3] Soifer M, Nguyen NV, Leite R, Fernandes J, Kodati S (2022). Recurrent Multiple Evanescent White Dot Syndrome (MEWDS) Following First Dose and Booster of the mRNA-1273 COVID-19 Vaccine: Case Report and Review of Literature. Vaccines.

[4] Baharani A, Reddy RR (2023). Multiple Evanescent White Dot Syndrome Following Adenovirus Vector-Based COVID-19 Vaccine (Covishield). Ocul Immunol Inflamm.

